# Supplementary material for: Heat adaptation of phage T7 under an extended genetic code
Source: Virus Evol. 2021 Dec 1;7(2):veab100. doi: 10.1093/ve/veab100 (PMC8923235; doi:10.1093/ve/veab100)
Supplement: veab100_Supp [file veab100_supp.zip › Sequencing_Analysis.zip!/Sequencing_Analysis/S2/evidence/RA_28.html]

BRESEQ :: Evidence


| Read alignment evidence... | | | | | | | | | | | |
| --- | --- | --- | --- | --- | --- | --- | --- | --- | --- | --- | --- |
|  | seq id | position | | ref | new | freq | score (cons/poly) | reads | annotation | genes | product |
| \* | NC\_001604.1 | 33,069 | 0 | G | T | 23.0% | 13164.9 / inf | 6771 | intergenic (–/–) | *–/–* | –/– |
| Reads supporting (aligned to +/- strand):  **ref** base G (2717/2491);  **new** base T (763/792);  **total** (3484/3287) | | | | | | | | | | | |
| Fisher's exact test for biased strand distribution *p*-value = 3.24e-02 | | | | | | | | | | | |
| Kolmogorov-Smirnov test that lower quality scores support variant *p*-value = 8.92e-01 | | | | | | | | | | | |

Only 100 of 6849 total aligned reads displayed.  

`GATGGTAGAGAAGTACGCTATGGATAAGGCTTATGGTATCTCCCACTCAGACCAGTTCACCAACAGTTCCATAATAGAAGAGAACATTGAGGGCTTAGTAGGTATCGAGAATAACTCATTCCTTGAGGCACGTAACTTGTTTGATTCGGACCTATCCATCACTATGCCAGACGGACAGCAATTCTCAGTGAATGACCTAAGGGACTTCGATATGTTCCGCA‑TCATGCCAGCGTATGACCGCCGTGTCAATGGTGACATCGCCATCATGGGGTCTACTGGTAAAACCACTAAGGAACTTAAGGATGAGATTTTGGCTCTCAAAGCGAAAGCTGAGGGAGACGGTAAGAAGACTGGCGAGGTACATGCTTTAATGGATACCGTTAAGATTCTTACTGGTCGTGCTAGACGCAATCAGGACACTGTGTGGGAAACCTCACTGCGTGCCATCAATGACCTAGGGTTCTTCGCTAAGAACGCCTACATGGGTGCTCAGAACATTACGGAGATTGCTGGGATGATTGTCACTGGTAACGTTCGTGCTCTAGGGCATGGTATCCCAATTCTGCGTGATACACTCTACAAGTCTAAACCAGTTTCAGCT  >  NC_001604.1/32769‑33369`  

                                                                                                                                                                                                                                                                                                             `|`                                                                                                                                                                                                                                                                                                              
`tataagagacaggTACGCTATGGATAAGGCTTATGGTATCTCCCACTCAGACCAGTTCACCAACAGTTCCATAATAGAAGAGAACATTGAGGGCTTAGTAGGTATCGAGAATAACTCATTCCTTGAGGCACGTAACTTGTTTGATTAGGACCTATCCATCACTATGCCAGACGGACAGCAATTCTCAGTGAATGACCTAAGGGACTTCGATATGTTCCGCA‑TCATGCCAGCGTATGACCGCCGTGTCAATGGTGGCATCGCCATCATGGGGTCTACTGGTAAAACCACTAAGGAACTTAAg                                                                                                                                                                                                                                                                                                              <  1:736052/289‑1 (MQ=255)`  
               `cGCTATGGATAAGGCTTATGGTATCTCCCACTCAGACCAGTTCACCAACCGTTCCATAATAGAAGAGAACATTGAGGGCTTAGTAGGTATCGAGAATAACTCATTCCTTGAGGCACGTAACTTGTTTGATTCGGACCTATCCATCACTATGCCAGACGGACAGCAATTCTCAGTGAATGACCTAAGGGACTTCGATATGTTCCGCA‑TCATGCCAGCGTATGACCGCCGTGTCAATGGTGACATCGCCATCATGGGGTCTACTGGTAAAACCACTAAGGAACTTAAgctgtctcttatacac                                                                                                                                                                                                                                                                                               >  1:700740/1‑286 (MQ=255)`  
                 `cTATGGATAAGGCTTATGGTATCTCCCACTCAGACCAGTTCACCAACAGTTCCATAATAGAAGAGAACACTGAGGGCTTAGTAGGTAGCGAGAATAACTCATTCCTTGAGGCACGTAACTTGTTTGAGTCGGACCTATCCATCACTATGCCAGACGGGCAGCAATTCTCAGTGAATGACCTAAGGGACTTCGATATGTTCCGCA‑TCATGCCAGCGTATGACCGCCGTGTCAATGGGGACATCGGCATCATGGAGTCTACGGGTAAAAACACTAAGGAACTTAAGGATGAGATTTTGGctct                                                                                                                                                                                                                                                                                             >  1:73008/1‑301 (MQ=255)`  
                   `aTGGATAAGGCTTATGGTATCTCCCACTCAGACCAGTTCACCAACAGTTCCATAAGAGAAGAGAACATTGAGGGCTTAGTAGGTATCGAGAATAACTCATTCCTTGAGGCACGTAACTTGTTTGATTCGGACCTATCCATCACTATGCCAGACGGACAGCAATTCTCAGTGACTGACCTAAGGGACTTCGATATGTTCCGCA‑TAATGCCAGCGTCTGACCGCCGTGTCAATGGTGACATCGCCATCATGGGGTCTACTGGTAAAACCACTAAGGAACTTAAGGATGAGATTTTGGCTCTCa                                                                                                                                                                                                                                                                                           >  1:808683/1‑301 (MQ=255)`  
                     `ggATAAGGCTTATGGTATCTCCCACTCAGACCAGTTCACCAACAGTTCCATAATAGAAGAGAACATTGAGGGCTTAGTAGGTATCGAGAATAACTCATTCCTTGAGGCACGTAACTTGTTTGATTCGGACCTATCCATCACTATGCCAGACGGACAGCAATTCTCAGTGAATGACCTAAGGGACTTCGATATGTTCCGCA‑TCATGCCAGCGTATGACCGCCGTGTCAATGGTGACATCGCCATCATGGGGTCTACTGGTAAAACCACTAAGGAACTTAATGATGAGATTTTGGcctgtctc                                                                                                                                                                                                                                                                                         >  1:278952/1‑294 (MQ=255)`  
                           `ggCTTATGGTATCTCCCACTCAGACCAGTTCACCAACAGTTCCATAATAGAAGAGAACATTGAGGGCTTAGTAGGTATCGAGAATAACTCATTCCTTGAGGCACGTAACTTGTTTGATTCGGACCTATCCATCACTATGCCAGGCGGACAGCAATTCTCAGTGAATGACCTAAGGGACTTCGATATGTTCCGCA‑TCATGCCAGCGTATGACCGCCGTGTCAATGGTGACATCGCCATCATGGGGTCTACTGGTAAAACCACTAAGGAACTTAATGATGAGATTTTGGCTCTCAAAGCGaaa                                                                                                                                                                                                                                                                                   >  1:1571/1‑301 (MQ=255)`  
                           `ggCTTATGGTATCTCCCACTCAGACCAGTTCACCAACAGTTCCATAATAGAAGAGAACATTGAGGGCTTAGTAGGTATCGAGAATAACTCATTCCTTGAGGCACGTAACTTGTTTGATTCGGACCTATCCATCACTATGCCAGACGGACAGCAATTCTCAGTGAATGACCTAAGGGACTTCGATATGTTCCGCA‑TCATGCCAGCGTATGACCGCTGTGTCAATGGTGACATCGCCATCATGGGGTCTACTGGTAAAACCACTAAGGAACTTAAGGATGAGATTTTGGCTCTCAAAGCGaaa                                                                                                                                                                                                                                                                                   >  1:6467/1‑301 (MQ=255)`  
                           `ggCTTATGGTATCTCCCACTCAGACCAGTTCACCAACAGTTCCATAATAGAAGAGAACATTGAGGGCTTAGTAGGTATCGAGAATAACTCATTCCTTGAGGCACGTAACTTGTTTGATTCGGACCTATCCATCACTATGCCAGACGGACAGCAATTCTCAGTGAATGACCTAAGGGACTTCGATATGTTCCGCA‑TCATGCCAGCGTATGACCGCCGTGTCAATGGTGACATCGCCATCATGGGGTCTACTGGTAAAACCACTAAGGAACTTAATGATGAGATTTTGGCTCTCAAAGCGaaa                                                                                                                                                                                                                                                                                   >  1:321294/1‑301 (MQ=255)`  
                                `aTGGTATCTCCCACTCAGACCAGTTCACCAACAGTTCCATAATAGAAGAGAACATTGAGGGCTTAGTAGGTATCGAGAATAACTCATTCCTTGAGGCACGTAACTTGTTTGATTCGGACCTATCCATCACTATGCCAGACGGACAGCAATTCTCAGTGAATGACCTAAGGGACTTCGATATGTTCCGCA‑TCATGCCAGCGTATGACCGCCGTGTCAATGGTGACATCGCCATCATGGGGTCTACTGGTAAAACCACTAAGGAACTTAATGATGAGATTTTGGCTCTCAAAGCGAAAGCTGa                                                                                                                                                                                                                                                                              >  1:183993/1‑301 (MQ=255)`  
                                            `aCTCAGACCAGTTCACCAACAGTTCCATAATAGAAGAGAACATTGAGGGCTTAGTAGGTATCGAGAATAACTCATTCCTTGAGGCACGTAACTTGTTTGATTCGGACCTATCCATCACTATGCCAGACGGACAGCAATTCTCAGTGAATGACCTAAGGGACTTCGATATGTTCCGCA‑TCATGCCAGCGTATGACCGCCGTGTCAATGGTGACATCGCCATCATTGGGTCTACTGGTAAAACCACTAAGGAACTTAATGATTAGATTTTGGCTCTCAAAGCGAAAGCTGAGGGAGACGGTaa                                                                                                                                                                                                                                                                  >  1:263974/1‑301 (MQ=255)`  
                                                 `gACCAGTTCACCAACAGTTCCATAATAGAAGAGAACATTGAGGGCTTAGTAGGTATCGAGAATAACTCATTCCTTGAGGCACGTAACTTGTTTGATTCGGACCTATCCATCACTATGCCAGACGGACAGCAATTCTCCGTGAATGACCTAAGGTACTTCGATATGTTCCGCA‑TCATGCCAGCGTATGACCGCCGTGTCAATGGTGGCATCGCCATCATGGGGTCTACTGGTAAAACCACTAAGGAACTTAAGGATGAGATTTTGGCTCTCAAAGCGAAAGCTGAGGGAGACGGTAagaaga                                                                                                                                                                                                                                                             >  1:328601/1‑301 (MQ=255)`  
                                                     `aGTTCACCAACAGTTCCATAATAGAAGAGAACATTGAGGGCTTAGTAGGTATCGAGAATAACTCATTCCTTGAGGCACGTAACTTGTTTGATTCGGACCTATCCATCACTATGCCAGACGGACAGCAATTCTCAGTGAATGACCTAAGGGACTTCGATATGTTCCGCA‑TCATGCCAGCGTATGACCGCCGTGTCAATGGTGGCATCGCCATCATGGGGTCTACTGGTAAAACCACTAAGGAACTTAAGGATGAGATTTTGGCTCTCAAAGCGAAAGCTGAGGGAGACGGTAAGAAGACTgg                                                                                                                                                                                                                                                         >  1:192597/1‑301 (MQ=255)`  
                                                      `gTTCACCAACAGTTCCATAATAGAAGAGAACATTGAGGGCTTAGTAGGTATCGAGAATAACTCATTCCTTGAGGCACGTAACTTGTTTGATTCGGACCTATCCATCACCATGCCAGACGGACAGCAATTCTCAGTGAATGACCTAAGGGACTTCGATATGTTCCGCA‑TCATGCCAGCGTATGACCGCCGTGTCAATGGTGACATCGCCATCATGGGGTCTACTGGTAAAACCACTAAGGAACTTAAGGATGAGATTTTGGCTCTCAAAGCGAAAGCTGAGGGAGACGGTAAGAAGACTggg                                                                                                                                                                                                                                                        >  1:492815/1‑300 (MQ=255)`  
                                                       `ttCACCAACAGTTCCATAATAGAAGAGAACATTGAGGGCTTAGTAGGTATCGAGAATAACTCATTCCTTGCGGAACGTAACTTGTTTGATTCGGACCTATCCATCACTATGCCAGACGGACAGCAATTCTCAGTGAATGACCTAAGGGACTTCGATATGTTCCGCA‑TCATGCCAGCGTATGACCGCAGTGTCAATGGTGACATCGCCATCATGGGGTCTACTGGTAAAACCACTAAGGAACTTAAGGATGAGATTTTGGCTCTCAAAGCGAAAGCTGAGGGAGACGGTAAGAAGACTGGCg                                                                                                                                                                                                                                                       <  1:472338/301‑1 (MQ=255)`  
                                                           `ccAACAGTTCCATAATAGAAGAGAACATTGAGGGCTTAGTAGGTATCGAGAATAACTCATTCCTTGAGGCACGTAACTTGTTTGATTCGGACCTATCCATCACTATGCCAGACGGACAGCAATTCTCAGTGAATGACCTAAGGGACTTAGATATGTTCCGCA‑TCATGCCAGCGTATGACCGCCGTGTCAATGGTAACATCGCCATCATGGGGTCTACTGGTAAAACCACTCAGGAACTTAAGGATGAGATTTTGGCTCTCAAAGCGAAAGCTGAGGGAGACGGTAAGAAGACTGGCGAGGt                                                                                                                                                                                                                                                   >  1:208242/1‑301 (MQ=255)`  
                                                               `cAGTTCCATAATAGAAGAGAACATTGAGGGCTTAGTAGGTATCGAGAATAACTCATTCCTTGAGGCACGTAACTTGTTTGATTCGGACCTATCCATCACTATGCCAGACGGACAGCAATTCTCAGTGAATGACCTAAGGGACTTCGATATGTTCCGCA‑TCATGCCAGCGTATGACCGCCGTGTCAATGGTGGCATCGCCATCATGGGGTCTACTGGTAAAACCACTAAGGAACTTAAGGATGAGATTTTGGCTCTAAAAGCGAAGGCTGAGGGAGACGGTaagaagcggcctcttataaaa                                                                                                                                                                                                                                               >  1:771476/1‑286 (MQ=255)`  
                                                                       `taataGAAGAGAACATTGAGGGCTTAGTAGGTATCGAGAATAACTCATTCCTTGAGGCACGTAACTTGTTTGATTCGGACCTATCCATCACTATGCCAGACGGACAGCAATTCTAAGTGAATGACCTAAGGGACTTCGATATGTTCCGCA‑TCATGCCAGCGTATGACCGCCGTGTCAATGGTGGCATCGCCATCATGGGGTCTACTGGTAAAACCACTAAGGAACTTAAGGATGAGATTTTGGCTCTCAAAGCGAAAGCTGAGGGAGACGGTAAGAAGACTGGCGAGGTACATGCTTTAAt                                                                                                                                                                                                                                       >  1:429226/1‑301 (MQ=255)`  
                                                                       `gtataagagacagcATTGAGGGCTTAGTAGGTATCGAGAATAACTCATTCCTTGAGGCACGTAACTTGTTTGATTCGGACCTATCCATCACTATGCCAGACGGACAGCAATTCTCAGTGAATGACCTAAGGGACTTCGATATGTTCCGCA‑TCATGCCAGCGTATGACCGCCGTGTCAATGGTGACATCGCCATCATGGGGTCTACTGGTAAAACCACTAAGGAACTTAATGATGAGATTTTGGCTCTCAAAGCGAAAGCTGAGGGAGACGGTAAGAAGACTGGCGAGGTACATGCTTTAAt                                                                                                                                                                                                                                       <  1:142739/288‑1 (MQ=255)`  
                                                                         `ataGAAGAGAACATTGAGGGCTTAGTAGGTATCGAGAATAACTCATTCCTTGAGGCACGTAACTTGTTTGATTCGGACCTATCCATCACTATGCCAGACGGACAGCAATTCTCAGTGAATGACCTAAGGGACTTCGATATGTTCCGCA‑TCATGCCAGCGTATGACCGCCGTGTCAATGGTGACATCGCCATCATGGGGTCTACTGGTAAAACAACTAAGGAACTTAAGGATGAGATTTTGGCTCTCAAAGCGAAAGCTGAGGGAGACGGTAAGAAGACTGGCGAGGTACATGCTTTAAtct                                                                                                                                                                                                                                     >  1:516512/1‑299 (MQ=255)`  
                                                                                    `cATTGAGGGCTTAGTAGGTATCGAGAATAACTCATTCCTTGAGGCACGTAACTTGTTTGATTCGGACCTATCCATCACTAAGCCAGACGGACAGCAATTCTCAGTGAATGACCTAAGGGACTTCGATATGTTCCGCA‑TCATGCCAGCGTATGACCGCCGTGTCAATGGTGACATCGCCATCATGGGGTCTACTGGTAAAACCACTAAGGAACTTAATGATGAGATTTTGGCTCTCAAAGCGAAAGCTGAGGGAGACGGTAAGAAGACTGGCGAGGTACATGCTTTAATGGATACCGTTAAg                                                                                                                                                                                                                          >  1:341984/1‑301 (MQ=255)`  
                                                                                    `cATTGAGGGCTTAGTAGGTATCGAGAATAACTCATTCCTTGAGGCACGTAACTTGTTTGATTCGGACCTATCAATCACTATGCCAGACGGACAGCAATTCTCAGTGAATGACCTAAGGGACTTCGATATGTTCCGCA‑TCATGCCAGCGTATGACCGCCGTGTCAATGGTGACATCGCCATCATGGGGTCTACTGGTAAAACCACTAAGGAACTTAAGGATGAGATTTTGGCTATCAAAGCGAAAGCTGAGGGAGACGGTAAGAAGACTGGCGAGGTACATGCTTTAATGGATACCGTTAAg                                                                                                                                                                                                                          >  1:55925/1‑301 (MQ=255)`  
                                                                                     `gacaggggCTTAGTAGGTATCGAGAATAACTCATTCCTTGAGGCACGTAACTTGTTTGATTCGGACCTATCCATCACTATGCCAGACGGACAGCAATTCTCAGTGAATGACCTAAGGGACTTCGATATGTTCCGCA‑TCATGCCAGCGTATGACCGCCGTGTCAATGGTGACATCGCCATCATGGGGTCTACTGGTAAAACCACTAAGGAACTTAATGATGAGATTTTGGCTCTCAAAGCGAAAGCTGAGGGAGACGGTAAGAAGACTGGCGAGGTACATGCTTTAATGGATACCGTTAAGa                                                                                                                                                                                                                         <  1:387383/296‑1 (MQ=255)`  
                                                                                     `aTTGAGGGCTTAGTAGGTATCGAGAATAACTCATTCCTTGAGGCACGTAACTTGTTTGATTCGGACCTATCCATCACTATGCCAGACGGACAGCAATTCTCAGTGAATGACCTAAGGGACTTCGATATGTTCCGCA‑TCATGCCAGCGTATGACCGCCGTGTCAATGGTGGCATCGCCATCATGGGGTCTACTGGTAAAACCACTAAGGAACTTAAGGATGAGATTTTGGCTCTCAAAGCGAAAGCTGAGGGAGACGGTAAGAAGACTGGCGAGGTACATGCTTTAATGGATACCGTTAAgt                                                                                                                                                                                                                         >  1:215589/1‑300 (MQ=255)`  
                                                                                     `aTTGAGGGCTTAGTAGGTATCGAGAATAACTCATTCCTTGAGGCACGTAACTTGTTTGATTCGGACCTATCCATCACTATGCCAGACGGACAGCAATTCTCAGGGAATGACCTAAGGGACTTCGATATGTTCCGCA‑TCATGCCAGCGTATGACCGCCGTGTCAATGGTGGCATCGCCATCATGGGGTCTACTGGTAAAACCACTAAGGAACTTAAGGATGAGATTTTGGCTCTCAAAGCGAAAGCTGAGGGAGACGGTAAGAAGACTGGCGAGGTACATGCTTTAATGGATACCGTTAAGa                                                                                                                                                                                                                         <  1:506713/301‑1 (MQ=255)`  
                                                                                     `aTTGAGGGCGTAGTAGGTATCGAGAATAACTCATTCCTTGAGGCACGTAACTTGTTTGATTCGGACCTATCCATCACTATGCCAGACGGACAGCAATTCTCAGTGAATGACCTAAGGGACTTCGATATGTTCCGCA‑TCATGCCAGCGTATGACCGCCGTGTCAATGGTGGCATCGCCATAATGGGGTCTACTGGTAAAACCACTAAGGAACTTAAGGATGAGATTTTGGCTCTCAAAGCGAAAGCTGAGGGAGACGGTAAGAAGACTGGCGAGGTACATGCTTTAATGGATACCGTTAAGa                                                                                                                                                                                                                         <  1:349024/301‑1 (MQ=255)`  
                                                                                      `ttGAGGGCTTAGTAGGTATCGAGAATAACTCATTCCTTGAGGCACGTAACTTGTTTGATTAGGACCTATCCATCACTATGCCAGACGGACAGCAATTCTCAGTGAATGACCTAAGTGACGTCGATATGTTCCGCA‑TCATGCCAGCGTATGACCGCCGTGTCAATGGTGCCATCGCCATCATGGGGTCTACTGGTAAAACCACTAAGGAACTTAAGGATGAGATTTTGGCTCTCAAAGCGAAAGCTGAGGGAGACGGTAAGAAGACTGGCGAGGTACATGCTTTAATGGATACCGTTAAGAt                                                                                                                                                                                                                        <  1:293438/301‑1 (MQ=255)`  
                                                                                       `tataagagacagagGTATCGAGAATAACTCATTCCTTGAGGCACGTAACTTGTTTGATTCGGACCTATCCATCACTATGCCAGACGGACAGCAATTCTCAGTGAATGACCTAAGGGACTTCGATATGTTCCGCA‑TCATGCCAGCGTATGACCGCCGTGTCAATGGTGACATCGCCATCATGGGGTCTACTGGTAAAACCACTAAGGAACTTAAGGATGAGA‑TTTGGCTCTCAAAGCGAAAGCTGAGGGAGACGGTAAGAAGACTGGCGAGGTACATGCTTTAATGGATACCGTTAAGATTc                                                                                                                                                                                                                      <  1:464748/289‑1 (MQ=255)`  
                                                                                        `tggggCTTAGTAGGTATCGAGAATAACTCATTCCTTGAGGCACGTAACTTGTTTGATTCGGACCTATCCATCACTATGCCAGACGGACAGCAATTCTCAGTGAATGACCTAAGGGACTTCGATATGTTCCGCA‑TCATGCCAGCGTATGACCGCCGTGTCAATGGTGACATCGCCATCATGGGGTCTACTGGTAAAACCACTAAGGAACTTAAGGATGAGATTTTGGCTCTCAAAGCGAAAGCTGAGGGAGACGGTAAGAAGACTGGCGAGGTACATGCTTTAATGGATACCGTTAAGATTc                                                                                                                                                                                                                      <  1:630205/299‑1 (MQ=255)`  
                                                                                            `gggagatgtgtataagagacagcTCATTCCTTGAGGCACGTAACTTGTTTGATTCGGACCTATCCATCACTATGCCAGACGGACAGCAATTCTCAGTGAATGACCTAAGGGACTTCGATATGTTCCGCA‑TCATGCCAGCGTATGACCGCCGTGTCAATGGTGACATCGCCATCATGGGGTCTACTGGTAAAACCACTAAGGAACTTAAGGATGAGATTTTGGCTCTCAAAGCGAAAGCTGAGGGAGACGGTAAGAAGACTGGCGAGGTACATGCTTTAATGGATACCGTTAAGATTCTTAc                                                                                                                                                                                                                  <  1:823740/279‑1 (MQ=255)`  
                                                                                            `gCTTAGTAGGTATCGAGAATAACTCATTCCTTGAGGCACGTAACTTGTTTGATTCGGACCTATCCATCACTATGCCAGACGGACAGCAATTCTCAGTGAATGACCTAAGGGACTTCGATATGTTCCGCA‑TCATGCCAGCGTATGACCGCCGTGTCAATGGTGACATCGCCATCATGGGGTCTACTGGTAAAACCACTAAGGAACTTAATGATGAGATTTTGGCTCTCAAAGCGAAAGCTGAGGGAGACGGTAAGAAGACTGGCGAGGTACATGCTTTAATGGATACCGTTAAGATTCTTAc                                                                                                                                                                                                                  >  1:248185/1‑301 (MQ=255)`  
                                                                                                  `gagGTATCGAGAATAACTCATTCCTTGAGGCACGTAACTTGTTTGATTCGGACCTATCCATCACTATGCCAGACGGACAGCAATTCTCAGTGAATGACCTAAGGGACTTCGATATGTTCCGCA‑TCATGCCAGCGTATGACCGCCGTGTCAATGGTGACATCGCCATCATGGGGTCTACTGGTAAAACCACTAAGGAACTTAAGGATGAGATTTTGGCTCTCAAAGCGAAAGCTGAGGGAGACGGTAAGAAGACTGGCGAGGTACATGCTTTAATGGATACCGTTAAGATTCTTACTGGTCg                                                                                                                                                                                                            >  1:53051/2‑301 (MQ=255)`  
                                                                                                      `tATCGAGAATAACTCATTCCTTGAGGCACGTAACTTGTTTGATTCGGACCTATCCATCACTATGCCAGACGGACAGCAATTCTCAGTGAATGACCTAAGGGACTTCGATATGTTCCGCA‑TCATGCCAGCGTATGACCGCCGTGTCAATGGTGGCATCGCCATCATGGGGTCTACTGGTAAAACCACTAAGGAACTTAAGGATGAGATTTTGGCTCTCAAAGCGAAAGCTGAGGGAGACGGTAAGAAGACTGGCGAGGTACATGCTTTAATGGATACCGTTAAGATTCTTACTGGTCGTGCt                                                                                                                                                                                                        <  1:638133/301‑1 (MQ=255)`  
                                                                                                                `aaCTCATTCCTTGAGGCACGTAACTTGTTTGATTCGGACCTATCCATCACTATGCCAGACGGACAGCAATTCTCAGTGAATGACCTAAGGGACTTCGATATGTTCCGCA‑TCATGCCAGCGTATGACCGCCGTGTCAATGGTGACATCGCCATCATGGGGTCTACTGGTAAAACCACTAAGGAACTTAATGATGAGATTTTGGCTCTCAAAGCGAAAGCTGAGGGAGACGGTAAGAAGACTGGCGAGGTACATGCTTTAATGGATACCGTTAAGATTCTTACTGGTCGTGCTAGACGCAATc                                                                                                                                                                                              <  1:599501/301‑1 (MQ=255)`  
                                                                                                                 `aCTCATTCCTTGAGGCACGTAACTTGTTTGATTCGGACCTATCCATCACTATGCCAGACGGACAGCAATTCTCAGTGAATGACCTAAGGGACTTCGATATGTTCCGCA‑TCATGCCAGCGTATGACCGCCGTGTCAATGGTGACATCGCCATCATGGGGTCTACTGGTAAAACCACTAAGGAACTTAAGGATGAGATTTTGGCTCTCAAAGCGAAAGCTGAGGGAGACGGTAAGAAGACTGGCGAGGTACATGCTTTAATGGATACCGTTAAGATTCTTACTGGTCGTGCTAGACGCAATCa                                                                                                                                                                                             >  1:592815/1‑301 (MQ=255)`  
                                                                                                                     `ggagatgtgtataagagacaggTTTGATTCGGACCTATCCATCACTATGCCAGACCGACAGCCATTCTCAGTGAATGACCTAAGGGACTTCGATATGTTCCGCA‑TCATGCCAGCGTATGACCGCCGTGTCAATGGTGACATCGCCATCATGGGGTCTACTGGTAAAACCACTAAGGAACTTAAGGATGAGATTTTGGCTCTCAAAGCGAAAGCTGAGGGAGACGGTAAGAAGACTGGCGAGGTACATGCTTTAATGGATACCGTTAAGATTCTTAGTGGTCGTGCTAGACGCAATCAGGac                                                                                                                                                                                         <  1:545304/280‑1 (MQ=255)`  
                                                                                                                       `tCCTTGAGGCACGTAACTTGTTTGATTCGGACCTATCCATCACTATGCCAGACGGACAGCAATTCTCAGTGAATGACCTAAGGGACTTCGATATGTTCCGCA‑TCATGCCAGCGTATGACCGCCGTGTCAATGGTGACATCGCCATCATGGGGTCTACTGGTAAAACCACTAAGGAACTTAAGGATGAGATTTCGGCTCTCAAAGCGAAAGCTGAGGGAGACGGTAAGAAGACTGGCGAGGTACATGCTTTAATGGATACCGTTAAGATTCTTACTGGTCGTGCTAGACGCAATCAGGacac                                                                                                                                                                                       <  1:486557/301‑1 (MQ=255)`  
                                                                                                                             `aGGCACGTAACTTGTTTGATTCGGACCTATCCATCACTATGCCAGACGGACAGCAATTCTCAGTGAATGACCTAAGGGACTTCGATATGTTCCGCA‑TCATGCCAGCGTATGACCGCCGTGTCAATGGTGGCATCGCCCTCATGGGGTCTACTGGTAAAACCACTAAGGAACTTAAGGATGAGATTTTGGCTCTCAAAGCGAAAGCTGAGGGAGACGGTAAGAAGACTGGCGAGGTACATGCTTTAATGGATACCGTTAAGATTCTTACTGGTCGTGCTAGACGCAATCAGGacacctgtct                                                                                                                                                                                 >  1:336235/1‑295 (MQ=255)`  
                                                                                                                               `tgtgtataagagacagATTCGGACCTCTCCATCACTATGCCAGACGGACAGCAATTCTCAGTGAATGACCTAATGGACTTCGATATGTTCCGCA‑TCATGCCAGCGTATGACCGCCGTGTCAATGGTAACATCGCCATCATGGGGTCTACTGGTAAAACCACTAAGGAACTTAAGGATGAGATTTTGGCTCTCAAAGCGAAAGCTGAGGGAGACGGTAAGAAGACTGGCGAGGTACATGCTTTAATGGATACCGTTAAGATTCTTACTGGTCGTGCTAGACGCAATCAGGACACTGTGTggg                                                                                                                                                                               <  1:743162/286‑1 (MQ=255)`  
                                                                                                                                 `acagaaCTTGTTTGATTCGGACCTATCCATCACTATGCCAGACGGACAGCAATTCTCAGTGAATGACCTAAGGGACTTCGATATGTTCCGCA‑TCATGCCAGCGTATGACCGCCGTGTCAATGGTGACATCGCCATCATGGGGTCTACTGGTAAAACCACTAAGGAACTTAATGATGAGATTTTGGCTCTCAAAGCGAAAGCTGAGGGAGACGGTAAGAAGACTGGCGAGGTACATGCTTTAATGGATACCGTTAAGATTCTTACTGGTCGTGCTAGACGCAATCAGGACACTGTGTGGGaa                                                                                                                                                                             <  1:623098/297‑1 (MQ=255)`  
                                                                                                                                 `aCGTAACTTGTTTGAATCGGACCTATCCATCACTATGCCAGACGGACAGCAATTCTCAGTGAATGACCTAAGGGACTTCGATATGTTCCGCA‑TCATGCCAGCGTATGACCGCCGTGTCAATGGTGGCATCGCCATCATGGGGTCTACTGGTAAAACCACTAAGGAACTTAAGGATGAGATTTTGGCTCTCAAAGCGAAAGCTGAGGGAGACGGTAAGAAGACTGGCGAGGTACATGCTTTAATGGATACCGTTAAGATTCTTACTGGTCGTGCTAGACGCAATCAGGACACTGTGTGGGaa                                                                                                                                                                             <  1:817147/301‑1 (MQ=255)`  
                                                                                                                                        `tttgttGATTCGGACCTAACCAACACTATGCTAGACGGACCACAATTCTCAGTGAATGACCTAAGGGACTTCGATATGTTCCGCA‑TCATGCCAGCGTATGACCGCCGTGTCAATGGTAACATCGCCATCATGGGGTCTACTGGTAAAACCACTAAGGAACTTAAGGATGAGATTTTGGCTCTCAAAACGAAAGCTGAGGGAGACGGTAAGAAGACTGGCGAGGTACATGCTTTAATGGATACCGTTAAGATTCTTACTGGTCGTGCTAGACGCAATCAGGACACTGTGTGGGAAACCTCAc                                                                                                                                                                      <  1:129235/297‑1 (MQ=255)`  
                                                                                                                                          `gTTTGATTCTGACCTAGCCATCACGATGCCAGACGGACAGCAATTCTCAGTGAATGACCTAAGGGATTTCGATATGTTCCGCA‑TCATGCCAGCGTATGACCGCCGTGTCAATGGTGACATCGCCATCATGGGGTCTACTGGTAAAACCACTAAGGAACTTAATGATGAGAGTTTGGCTCTCAAAGCGAAAGCTGAGGGAGACGGTAAGAAGACTGGCGAGGTACATGCTTTAATGGATACCGTTAAGATTCTTACTGGTCGTGCTAGACGCAATCAGGACACTGTGTGGGAAACCTAACTg                                                                                                                                                                    <  1:149998/301‑1 (MQ=255)`  
                                                                                                                                               `aTTCGGACCTATCCATCACTATGCCAGACGGACAGCAATTCTCAGTGAATGACCTAAGGGACTTCGATATGTTCCGCA‑TCATGCCAGCGTATGACCGCCGTGTCAATGGTGACATCGCCATCATGGGGTCTACTGGTAAAACCACTAAGGAACTTAAGGATGAGATTTTGGCTCTCAAAGCGAAAGCTGAGGGAGACGGTAAGAAGACTGGCGAGGTACATGCTTTAATGGATACCGTTAAGATTCTTACTGGTCGTGCTAGACGCAATCAGGACACTGTGTGGGAAACCTCACTGCGTGc                                                                                                                                                               >  1:307459/1‑301 (MQ=255)`  
                                                                                                                                                  `aggACCTATCCATCACTATTCCAGACGGACAGCAATTCTCAGTGAATGACCTAAGGGACTTCGATATGTTCCGCA‑TCATGCCAGCGTATGACCGCCGTGTCAATGGTGACATCGCCATCATGGGGTCTACTGGTAAAACCACTAAGGAACTTAAGGATGAGATTTTGGCTCTCAAAGCGAAAGCTGAGGGAGACGGTAAGAAGACTGGCGAGGTACATGCTTTAATGGATACCGTTAAGATTCTTACTGGTCGTGCTAGACGCAATCAGGACACTGTGTGGGAAACCTCACTGCGTGCCAt                                                                                                                                                            <  1:686054/300‑1 (MQ=255)`  
                                                                                                                                                    `gACCTATCCATCACTATGCCAGACGGACAGCAATTCTCAGTGAATGACCTAAGGGACTTCGATATGTTCCGCA‑TCATGCCAGCGTATGACCGCCGTGTCAATGGTGACATCGCCATCATGGGGTCTACTGGTAAAACCACTAAGGAACTTAAGGATGAGATTTTGGCTCTCAAAGCGAAAGCTGAGGGAGACGGTAAGAAGACTGGCGAGGTACATGCTTTAATGGATACCGTTAAGATTCTTACTGGTCGTGATAGACGCAATCAGGACACTGTGTGGGAAACCTCACTGCGTGCCATCa                                                                                                                                                          >  1:758288/1‑301 (MQ=255)`  
                                                                                                                                                      `cagaTCCATCACCATGCCAGACGGACAGCAATTCTCAGTGAATGACCTAAGGGACTTCGATATGTTCCGCA‑TCATGCCAGCGTATGACCGCCGTGTCAATGGTGACATCGCCATCATGAGATCTACTGGTAAAACCACTAAGGAACTTAAGGATGAGATTTTGGCTCTCAAAGCGAAAGCTGAGGGAGACGGTAAGAAGACTGGCGAGGTACATGCTTTAATGGATACCGTTAAGATTCTTACTGGTCGTGCTAGACGCAATCAGGACACTGTGTGGGAAACCTCACTGCGTGCCATCAAt                                                                                                                                                        <  1:793626/298‑1 (MQ=255)`  
                                                                                                                                                       `cTATCCATCACTATGCCAGACGGACATCAATTCTCAGTGAATGACCTAGCGGACTTCGATATGTTCCGCA‑TCATGCCAGCGTATGACCGCCGTGTCAATGGTGACATCGCCATCATGGGGTCTACTGGTAAAACCACTAAGGAACTTAAGGATGAGATTTTGCCTCTCAAAGCGAAAGCCGAGGTAGACGGTAAGAAGACTGGCGAGGTACATGCTTTAATGGTAACCGTTACGCTGCTTACTGGTCTTGCTATACGCAATCAGGAAACTGTGTGGGAAACCCCCCTGCGTGCCATCAATg                                                                                                                                                       >  1:356118/1‑301 (MQ=255)`  
                                                                                                                                                         `aTCCATCACTATGCCAGACGGACAGCAATTCTCAGTGAATGACCTAAGGGACTTCGATATGTTCCGCA‑TCATGCCAGCGTATGACCGCCGTGTCAATGGTGGCATCGCCATCATGGGGTCTACTGGTAAAACCACTAAGGAACTTAAGGATGAGATTTTGGCTCTCAAAGCGAAAGCTGAGGGAGACGGTAAGAAGACTGGCGAGGTACATGCTTTAATGGATACCGTTAAGATTCTTACTGGTCGTGCTAGACGCAATCAGGACACTGTGTGGGAAACCTCACTGCGTGCCATctgtctc                                                                                                                                                     >  1:421241/1‑295 (MQ=255)`  
                                                                                                                                                         `aTCCATCACTATGCCAGACGGACAGCAATTCTCAGTGAATGACCTAAGGGACTTCGATATGTTCCGAA‑TCATGCCAGCGTATGACCGACGTGTCAATGGTGACATCGCCATCATGTGGTCTACTGGTAAAACCACTAAGGAACTTAATGATGAGATTTTGGCTCTCAAAGCGAAAGCTGAGGGAGACGGTAAGAAGACTGGCGAGGTACATGCTTTAATGGATACCGTTAAGATTCTTACTGGTCGTGCTAGACGCAATCAGGACACTGTGTGGGAAACCTCACTGCGTGCCATCAATGAc                                                                                                                                                     >  1:136751/1‑301 (MQ=255)`  
                                                                                                                                                          `tCCATCACTATGCCAGACGGACAGCAATTCTCAGTGAATGACCTAAGGGACTTCGATATGTTCCGCA‑TCATGCCAGCGTATGACCGCCGTGTTAATGGTGACATCGCCATCATGGGGTCTACTGGTAAAACCACTAAGGAACTTAAGGATGAGATTTTGGCTCTCAAAGCGAAAGCTGAGGGAGACGGTAAGAAGACTGGCGAGGTACATGCTTTAATGGATACCGTTAAGATTCTTACTGGTCGTGCTAGACGCAATCAGGACACTGTGTGGGAAACCTCACTGCGTGCCATCAATGAcc                                                                                                                                                    <  1:616022/301‑1 (MQ=255)`  
                                                                                                                                                          `tCCATCACTATGCCAGACGGACAGCAATTCTCAGTGAATGACCTAAGGGACTTCGATATGTTCCGCA‑TCATGCCAGCGTATGACCGCCGTGTCAATGGTGACATCGCCATCATGGGGTCTACTGGTAAAACCACTAAGGAACTTAAGGATGAGATTTTGGCTCTCAAAGCGAAAGCTGAGGGAGACGGTAAGAAGACTGGCGAGGTACATGCTTTAATGGATACCGTTAAGATTCTTACTGGTCGTGCTAGACGCAATCAGGACACTGTGTGGGAAACCTCACTGCGTGCCATCAATGAcc                                                                                                                                                    <  1:313580/301‑1 (MQ=255)`  
                                                                                                                                                            `cATCACTATGCCAGACGGACAGCAATTCTCAGTGAATGACCTAAGGGACTTCGATATGTTCCGCA‑TCATGCCAGCGTATGACCGCCGTGTCAATGGTGACATCGCCATCATGGGGTCTACTGGTAAAACCACTAAGGAACTTAATGATGAGATTTTGGCTCTCAAAGCGAAAGCTGAGGGAGACGGTAAGAAGACTGGCGAGGTACATGCTTTAATGGATACCGTTAAGATTCTTACTGGTCGTGCTAGACGCAATCAGGACACTGTGTGGGAAACCTCACTGCGTGCCATCAATGACCTa                                                                                                                                                  <  1:363570/301‑1 (MQ=255)`  
                                                                                                                                                              `tCACTATGCCTGACGGACAGCAATTCTCAGTGAATGACCAAAGGGACTTCGATATGTTCCGCA‑TCATGCCAGCGTATGACCGCCGTGTCAATGGTAACATCGCCATCATGGGGTCTACTGGTAAAACCACTAAGGAACTTAAGGATGAGATTTTGGCTCTCAAAGCGAAAGCTGAGGGAGACGGTAAGAAGACTGGCGAGGTACATGCTTTAATGGATACCGTTAAGATTCTTACTGGTCGTGCTAGACGCAATCAGGACACTGTGTGGGAAACCTCACTGCGTGCCATCAATGACCTAgg                                                                                                                                                >  1:663068/1‑301 (MQ=255)`  
                                                                                                                                                               `cACTATGCCAGACGGACAGCAATTCTCAGTGAATGACCTAAGGGACTTCGATATGTTCCGCA‑TCATGCCAGCGTATGACCGCCGTGTCAATGGTGACATCGCCATCATGGGGTCTACTGGTAAAACCACTAAGGAACTTAAGGATGAGATTTTGGCTCTCAAAGCGAAAGCTGAGGGAGACGGTAAGAAGACTGGCGAGGTACATGCTTTAATGGATACCGTTAAGATTCTTACTGGTCGTGCTAGACGCAATCAGGACACTGTGTGGGAAACCTCACTGCGTGCCATCAATGACCTAggg                                                                                                                                               >  1:437169/1‑301 (MQ=255)`  
                                                                                                                                                               `cACTATGCCAGACGGACAGCAATTCTCAGTGAATGACCTAAGGGACTTCGATATGTTCCGCA‑TCATGCCAGCGTATGACCGCCGTGTCAATGGTGACATCGCCATCATGGGGTCTACTGGTAAAACCACTAAGGAACTTAAGGATGAGATTTTGGCTCTCAAAGCGAAAGCTGAGGGAGACGGTAAGAAGACTGGCGAGGTACATGCTTTAATGGATACCGTTAAGATTCTTACTGGTCGTGCTAGACGCAATCAGGACACTGTGTGGGAAACCTCACTGCGTGCCATCAATGACCTAggg                                                                                                                                               <  1:413604/301‑1 (MQ=255)`  
                                                                                                                                                                `aaaaTGCCAGACGGACAGCAATTCTCAGTGAAGGACCTAAGGAACTTCGATATGTTCCGCA‑TCATGCCAGCGTATGACCGCCGTGTCAATGGTAACATCGCAATCATGGGGTCTACGGGTAAAACCACAAAGGAACTTAAGGATGAGATTTTGGCTCTAAAAGCGAAAGCTGAGGGAGACGGTAAGAAGACTGGCGAGGAACATGCTTTAATAGATACCGTTAAGATTCTTACTGGTCGTGCTAGACGCAATCAGGACACTGTGTGGGAAACCTCACTGCGTGCCATCAATGACCTAGGGt                                                                                                                                              <  1:369952/298‑1 (MQ=255)`  
                                                                                                                                                                `aCTATGCCAGACGGACAGCAATTCTCAGTGAATGACCTAAGGGACTGCGATATGTTCCGCA‑TCATGCCAGCGTATGACCGCCGTGTCAATGGTGACATCGCCATCATGGGGTCTACTGGTAAAACCACTAAGGAACTTAATGATGAGATTTTGGCTCTCAAAGCGAAAGCTGAGGGAGACGGTAAGAAGACTGGCGAGGTACATGCTTTAATGGATACCGTTAAGATTCTTACTGGTCGTGCTAGACGCAATCAGGACACTGTGTGGGAAACCTCACTGCGTGCCATCAATGACCTAGGGt                                                                                                                                              <  1:378424/301‑1 (MQ=255)`  
                                                                                                                                                                   `aTGCCAGACGGACAGCAATTCTCAGTGAATGACCTAAGGGACTTCGATATGTTCCGCA‑TCATGCCAGCGTATGACCGCCGTGTCAATGGTGACATCGCCATCATGGGGTCTACTGGTAAAACCACTAAGGAACTTAAGGATGAGATTTTGGCTCTCAAAGCGAAAGCTGAGGGAGACGGTAAGAAGACTGGCGAGGTACATTCTTTAATGTATACCGTTAAGATTCTTACTGGTCGTGCTAGACGCAATCAGGACACTGTGTGGGAAACCTCACTGCGTGCCATCAATGAcactggctctt                                                                                                                                           >  1:174522/1‑291 (MQ=255)`  
                                                                                                                                                                      `ccAGACGGACAGCAATTCTCAGTGAATGACCTAAGGGACTTCGATATGTTCCGCA‑TCATGCCAGCGTATGACCGCCGTGTCAATGGTGACATCGCCATCATGGGGTCTACTGGTACAACCACTAAGGAACTTAATGATGAGATTTTGGCTCTCAAAGCGAAAGCTGAGGGAGACGGTAAGAAGACTGGCGAGGTACATGCTTTAATGGATACCGTTAAGATTCTTACTGGTCGTGCTAGACGCAATCAGGACACTGTGTGGGAAACCTCACTGCGTGCCATCAATGACCTAGGGtctgtcc                                                                                                                                        >  1:679875/1‑295 (MQ=255)`  
                                                                                                                                                                       `cAGACGGACAGCAATTCTCAGTGAATGACCTAAGGGACTTCGATATGTTCCGCA‑TCATGCCAGCGTATGACCGCCGTGTCAATGGTGGCATCGCCATCATGGGGTCTACTGGTAAAACCACTAAGGAACTTAAGGATGAGATTTTGGCTCTCAAAGCGAAAGCTGAGGGAGACGGTAAGAAGACTGGCGAGGTACATGCTTTAATGGATACCGTTAAGATTCTTACTGGTCGTGCTAGACGCAATCAGGACACTGTGTGGGAAACCTCACTGCGTGCCATCAATGACCTAGGGTTCTTCGc                                                                                                                                       <  1:523228/301‑1 (MQ=255)`  
                                                                                                                                                                       `cAGACGGACAGCAATTCTAAGTGAATGAACTAAGGGACTTCGATATGTTCCGCA‑TCATGCCAGCGTAAGACCGCCGTGTCAATGGTGACATCGCCATCATGGGGTCTACTGGTAAAACCACTAAGGAACTTAAGGATGAGATTTTGGCTCTCAAAGCGAAAGCTGAGGGAGACGGTAAGAAGACTGGCGAGGTACATGCTTTAATGGATACCGTTAAGATTCTTACTGGTCGTGCTAGACGCAATCAGGACACTGTGTGGGAAACCTCACTGCGTGCCATCAATGACCTAGGGTTCTTCGc                                                                                                                                       <  1:606685/301‑1 (MQ=255)`  
                                                                                                                                                                                 `gCAATTCTCAGTGAATGACCTAAGGGACTTCGATATGTTCCTCA‑TCATGCCAGCGTATGACCGCCGTGTCAATGGTGACATCGCCATCATGGGGTCTACTGGTAAAACCACTAAGGAACTTAAGGATGAGATTTTGGCTCTCAAAGCGAAAGCTGAGGGAGACGGTAAGAAGACTGGCGAGGTACATGCTTTAATGGATACCGTTAAGATTCTTACTGGTCGTGCTAGACGCAATCAGGACACTGTGTGGGAAACCTCACTGCGTGCCATCAATGACCTAGGGTTCTTCGCTAAGAACGcc                                                                                                                             >  1:552525/1‑301 (MQ=255)`  
                                                                                                                                                                                 `gCAATTCTCAGTGAATGACCTAAGGGACTTCGATATGTTCCGCA‑TCATGCCAGCGTATGACCGCCGTGTCAATGGTGACATCGCCATCATGGGGTCTACTGGTAAAACCACTAAGGAACTTAATGATGAGATTTTGGCTCTCAAAGCGAAAGCTGAGGGAGACGGTAAGAAGACTGGCGAGGTACATGCTTTAATGGATACCGTTAAGATTCTTACTGGTCGTGCTAGACGCAATCAGGACACTGTGTGGGAAACCTCACTGCGTGCCATCAATGACCTAGGGGTCTTCGCTAAGAACGcc                                                                                                                             >  1:722923/1‑301 (MQ=255)`  
                                                                                                                                                                                    `aTTCTCAGTGAATGACCTAAGGGACTTCGATATGTTCCGCA‑TCATGCCAGCGTATGACCGCCGTGTCAATGGTGACATCGCCATCATGGGGTCTACTGGTAAAACCACTAAGGAACTTAAGGATGAGATTTTGGCTCTCAACGCGAAAGCTGAGGGAGACGGTAAGAAGACTGGCGAGGTACATGCTTTAATGGATACCGTTAAGATTCTTACTGGTCGTGCTAGACGCAATCAGGACACTGTGTGGGAAACCTCACTGCGTGCCATCAATGACCTAGGGTTCTTCGCTAAGAACGCATaa                                                                                                                          >  1:714893/1‑300 (MQ=255)`  
                                                                                                                                                                                      `tctcAGTGAATGACCTAAGGGACTTCGATATGTTCCGCA‑TCATGCCAGCGTATGACCGCCGTGTCAATGGTGACATCGCCATCATGGGGTCTACTGGTAAAACCACTAAGGAACTTAAGGATGAGATTTTGGCTCTCAAAGCGAAAGCTGAGGGAGACGGTAAGAAGACTGGCGAGGTACATGCTTTAATGGATACCGTTAAGATTCTTACTGGTCGTGCTAGACGTAATCAGGACACTGTGTGGGAAACCTCACTGCGTGCCATCAATGACCTAGGGTTCTTCGCTAAGAACGCCTACAt                                                                                                                        <  1:500357/301‑1 (MQ=255)`  
                                                                                                                                                                                      `ggagatgtgtataagagacagACTTCGATATGTTCCGCA‑TCATGCCAGCGTATGACCGCCGTGTCAATGGTGACATCGCCATCATGGGGTCTACTGGTAAAACCACTAAGGAACTTAAGGATGAGATTTTGGCTCTCAAAGCGAAAGCTGAGGGAGACGGTAAGAAGACTGGCGAGGTACATGCTTTAATGGATACCGTTAAGATTCTTACTGGTCGTGCTAGACGCAATCAGGACACTGTGTGGGAAACCTCACTGCGTGCCATCAATGACCTAGGGTTCTTCGCTAAGAACGCCTACAt                                                                                                                        <  1:285302/281‑1 (MQ=255)`  
                                                                                                                                                                                      `gacaggTGAATGACCTAAGGGACTTCGATATGTTCCGCA‑TCATGCCAGCGTATGACCGCCGTGTCAATGGTGACATCGCCATCATGGGGTCTACTGGTAAAACCACTAAGGAACTTAAGGATGAGATTTTGGCTCTCAAAGCGAAAGCTGAGGGAGACGGTAAGAAGACTGGCGAGGTACATGCTTTAATGGATACCGTTAAGATTCTTACTGGTCGTGCTAGACGCAATCAGGACACTGTGTGGGAAACCTCACTGCGTGCCATCAATGACCTAGGGTTCTTCGCTAAGAACGCCTACAt                                                                                                                        <  1:232623/296‑1 (MQ=255)`  
                                                                                                                                                                                         `cAGTTAAGGACCTAAGGGACTTCGACATGTCCCCCAGCCATG‑CAGCGTATGACCCCCGTGTCAATGGTGGCATCGCCATCATGTGGTCTACTGGTAAAACCACTAAGGAACTTAAGGATGAGATTTTGGCTCTCAAAGCGAAAGCTTAGGGAGACGGTAAGAAGCCTGGCGAGGTACATGCTTTAATGGATACCGTTAAGATTCTTACCGGTCGTGCTAGACGCAATCAGGACACTGTTTGGGAAACCTCACTGCGTGCCATCAATGACCTAGGGTTCTTCGCTAAGACCGCCTACATggg                                                                                                                     <  1:107046/301‑1 (MQ=255)`  
                                                                                                                                                                                         `aggTGAATGACCTAAGGGACTTCGATATGTTCCGCA‑TCATGCCAGCGTATGACCGCCGTGTCAATGGTGACATCGCCATCATGGGGTCTACTGGTAAAACCACTAAGGAACTTAAGGATGAGATTTTGGCTCTCAAAGCGAAAGCTGAGGGAGACGGTAAGAAGACTGGCGAGGTACATGCTTTAATGGATACCGTTAAGATTCTTACTGGTCGTGCTAGACGCAATCAGGACACTGTGTGGGAAACCTCACTGCGTGCCATCAATGACCTAGGGTTCTTCGCTAAGAACGCCTACATggg                                                                                                                     <  1:671937/299‑1 (MQ=255)`  
                                                                                                                                                                                             `gAATGACCTAAGGGACTTCGATATGTTCCGCA‑TCATGCCAGCGTATGACCGCCGTGTCAATGGTGACATCGCCATCATGGGGTCTACTGGTAAAACCACTAAGGAACTTAAGGATGAGATTTTGGCTCTCAAAGCGAAAGCTGAGGGAGACGGTAAGAAGACTGGCGAGGTACATGCTTTAATGGATACCGTTAAGATTCTTACTGGTCGTGCTAGACGCAATCAGGACACTGTGTGGGAAACCTCACTGCGTGCCATCAATGACCTAGGGTTCTTCGCTAAGAACGCCTACAGGGCTgtg                                                                                                                 >  1:240762/1‑299 (MQ=255)`  
                                                                                                                                                                                               `aTGACCTAAGGGACTTCGATATGTTCCGCA‑TCATGCCAGCGTATGACCGCCGTGTCAATGGTGACATCGCCATCATGGGGTCTACTGGTAAAACCACTAAGGAACTTAAGGATGAGATTTTGGCTCTCAAAGCGAAAGCTGAGGGAGACGGTAAGAAGACTGGCGAGGTACA‑‑‑‑‑‑‑‑‑‑‑‑‑‑‑‑‑‑‑‑‑‑‑‑TCTTACTGGTCGTGCTAGACGCAATCAGGACACTGTGTGGGAAACCTCACTGCGTTCCATCAATGACCTAGGGTTCTTCGCTAAGAACGCCTACATGGGTGCTCAGAACATTACGGAGATTGCCGGgag                                                                                       >  1:585194/1‑300 (MQ=255)`  
                                                                                                                                                                                                 `gACCTAAGGGACTTCGATATGTTCCGCA‑TCATGCCAGCGTATGACCGCCGTGTCAATGGTGACATTGCCATCATGGGGTCTACTGGTAAAACCACTAAGGAACTTAAGGATGAGATTTTGGCTCTCAATGCGAAAGCTGAGGGAGCCGGTAAGAAGACTGGCGAGGTACATGCTTTAACGGCTACCGTTAAGATTCTTACTGGTCGTGCTAGACGCAATAAGGACACTGTGTGGGAAACCTCACTGCGTGCCATCAATAAGCTAGGGCTCTTCGCTAAGAACGCCTACTTGGTTGCTCAGa                                                                                                             >  1:538322/1‑301 (MQ=255)`  
                                                                                                                                                                                                  `aCCTAAGGGACTTCGATATGTTCCGCA‑TCATGCCAGCGTATGACCGCCGTGTCAATGGTGGCATCGCCATCATGGGGTCTACTGGTAAAACCACTAAGGAACTTAAGGATGAGATTTTGGCTCTCAAAGCGAAAGCTGAGGGAGACGGTAAGAAGACTGGCGAGGTACATGCTTTAATGGATACCGTTAAGATTCTTACTGGTCGTGCTAGACGCAATCAGGACACTGTGTGGGAAACCTCACTGCGTGCCATCAATGACCTAGGGTTCTTCGCTAAGAACGCCTACATgggctgtctctt                                                                                                            >  1:653646/1‑292 (MQ=255)`  
                                                                                                                                                                                                     `tAAGGGACTTCGATATGTTCCGCA‑TCATGCCAGCGTATGACCGCCGTGTCAATTGTGACATCGCCATCATGGTGTCTACTGGTAAAACCACTAAAGAACTTAAGGATGAGATTTTGGCTCTCAAAGCGAAAGCTGAGGGAGACGGTAAGAAGACTGGCGAGGTACATGCTTTAATGGATACCGTTAAGATTCTTACTGGTCGTGCTAGACGCAATCAGGACACTGTGTGGGAAACCTCACTGCGTGCCATCAATGACCTAGGGTTCTTCGCTAAGAACGCCTACATGGGTGCTCAGAACAt                                                                                                         <  1:786133/301‑1 (MQ=255)`  
                                                                                                                                                                                                        `gggACTTCGATATGTTCCGCA‑TCATGCCAGCGTATGACCGCCGTGTCAATGGTGGCATCGCCATCATGGGGTCTACTGGTAAAACCACTAAGGAACTTAAGGATGAGATTTTGGCTCTCAAAGCGAAAGCTGAGGGAGACGGTAAGAAGACTGGCGAGGTACATGCTTTAATGGATACCGTTAAGATTCTTACTGGTCGTGCTAGACGCAATCAGGACACTGTGTGGGAAACCTCACTGCGTGCCATCAATGACCTAGGGTTCTTCGCTAAGAACGCCTACATGGGTGCTCAGAACATTAc                                                                                                      <  1:480422/301‑1 (MQ=255)`  
                                                                                                                                                                                                          `aagagacagatGTTCCGCA‑TCATGCCAGCGTATGACCGCCGTGTCAATGGTGGCATCGCCATCATGGGGTCTACTGGTAAAACCACTAAGGAACTTAAGGATGAGATTTTGGCTCTCAAAGCGAAAGCTGAGGGAGACGGTAAGAAGACTGGCGAGGTACATGCTTTAATGGATACCGTTAAGATTCTTACTGGTCGTGCTAGACGCAATCAGGACACTGTGTGGGAAACCTCACTGCGTGCCATCAATGACCTAGGGTTCTTCGCTAAGAACGCCTACATGGGTGCTCAGAACATTACgg                                                                                                    <  1:113642/292‑1 (MQ=255)`  
                                                                                                                                                                                                              `tCGCTATGTTCCGCA‑TCATGCCAGCGTATGACCGCCGTGTCAATGGTGACATCGCCATCATGGGGTCTACTGGTAAAACCACTAAGGAACTTAATGATGAGATTTTGGCTCTCAAAGCGAAAGCTGAGGGAGACGGTAAGAAGACTGGCGAGGTACATGCTTTAATGGATACCGTTAAGATTCTTACTGGTCGTGCTAGACGCAATCAGGACACTGTGTGGGAAACCTCACTGCGTGCCATCAATGACCTAGGGTTCTTCGCTAAGAACGCCTACATGGGTGCTCAGactgtctcttatac                                                                                                >  1:271197/1‑288 (MQ=255)`  
                                                                                                                                                                                                              `tCGATATGTTCCGCA‑TCATGCCAGCGTATGACCGCCGTGTCAATGGTGACATCGCCATCATGGGGTCTACTGGTAAAACCACTAAGGAACTTAAGGATGAGATTTTGGCTCTCAAAGCGAAAGCTGAGGGAGACGGTAAGAAGACTGGCGAGGTACATGCTTTAATGGATACCGTTAAGATTCTTACTGGTCGTGCTAGACGCAATCAGGACACTGTGTGGGAAACCTCACTGCGTGCCATCAATGACCTAGGGTTCTTCGCTAAGAACGCCTACATGGGTGCTCAGAACATTACGGAGAt                                                                                                >  1:300340/1‑301 (MQ=255)`  
                                                                                                                                                                                                                     `gTTCCGCA‑TCATGCCAGCGTATGACCGCCGTGTCAATGGTGGCATCGCCATCATGGGGTCTACTGGTAAAACCACTAAGGAACTTAAGGATGAGATTTTGGCTCTCAAAGCGAAAGCTGAGGGAGACGGTAAGAAGACTGGCGAGGTACATGCTTTAATGGATACCGTTAAGATTCTTACTGGTCGTGCTAGACGCAATCAGGACACTGTGTGGGAAACCTCACTGCGTGCCATCAATGACCTAGGGTTCTTCGCTAAGAACGCCTACATGGGTGCTCAGAACATTACGGAGATTGCTggg                                                                                         >  1:122216/1‑301 (MQ=255)`  
                                                                                                                                                                                                                     `gTTCCGCA‑TCATGCCAGCGTATGACCGCCGTGTCAATGGTGACATCGCCATCATGGGGTCTACTGGTAAAACCACTAAGGAACTTAAGGATGAGATTTTGGCTCTCAAAGCGAAAGCTGAGGGAGACGGTAAGAAGACTGGCGAGGTACATGCTTTAATGGATACCGTTAAGATTCTTACTGGTCGTGCTAGACGCAATCAGGACACTGTGTGGGAAACCTCACTGCGTGCCATCAATGACCTAGGGTTCTTCGCTAATAACGCCTACATGGGTGCTCAGAACATTACGGAGACTgtctct                                                                                         >  1:800314/1‑296 (MQ=255)`  
                                                                                                                                                                                                                     `gTTCCGCA‑TCATGCCAGCGTATGACCGCCGTGTCAATGGTGACATCGCCATCATGGGGTCTACTGGTAAAACCACTAAGGAACTTAAGGATGAGATGTTGGCTCTCAAAGCGAAAGCTGAGGGAGACGGTAAGAAGACTGGCGAGGTACATGCTTTAATGGATACCGTTAAGATTCTTACTGGTCGTGCTAGACGCAATCAGGACACTGTGTGGGTAACCTCACTGCGTGCCATCAATGACCTAGGGTTCTTCGCTAAGAACGCCTACATGGGTGCTCAGAACATTACGGAGATTGCCggg                                                                                         >  1:39371/1‑301 (MQ=255)`  
                                                                                                                                                                                                                           `ca‑tcatGCCAGCGTATGACCGCCGTGTCAATGGTGGCATCGCCATCATGGGGTCTACTGGTAAAACCACTAAGGAACTTAAGGATGAGATTTTGGCTCTCAAAGCGAAAGCTGAGGGAGACGGTAAGAAGACTGGCGAGGTACATGCTTTAATGGATACCGTTAAGATTCTTACTGGTCGTGCTAGACGCAATCAGGACACTGTGTGGGAAACCTCACTGCGTGCCATCAATGACCTAGGGTTCTTCGCTAAGAACGCCTACATGGGTGCTCAGAACATTACGGAGATTGCTGGGATGAtt                                                                                   >  1:707230/1‑301 (MQ=255)`  
                                                                                                                                                                                                                            `a‑tcatGCCAGCGTATGACCGCCGTGTCAATGGTGACATCGCCATCATGGGGTCTACTGGTAAAACCACTAAGGAACTTAAGGATGAGATTTTGGCTCTCAAAGCGAAATCTGAGGGAGACGGTAAGAAGACTGGCGAGGTACATGCTTTAATGGATACCGTTAAGATTCTTACTGGTCGTGCTAGACGCAATCAGGACACTGTGTGGGAAACCTCACTGCGTGCCATCAATGACCTAGGGTTCTTCGCTAAGAACGCCTACATGGGTGCTCAGAACATTACGGAGATAGCCGGGATGATTg                                                                                  <  1:445400/301‑1 (MQ=255)`  
                                                                                                                                                                                                                              `tcatGCCAGCGTATGACCGCCGTGTCAATGGTGACATCGCCATCATGGGGTCTACTGGTAAAACCACTAAGGAACTTAAGGATGAGATTTTGGCTCTCAAAGCGAAAGCTGAGGGAGACGGTAAGAAGACTGGCGAGGTACATGCTTTAATGGATACCGTTAAGATTCTTACTGGTCGTGCTAGACGCAATCAGGACACTGTGTGGGAAACCTCACTGCGTGCCATCAATGACCTCGGGTTCTTCGCTAAGAACGCCTACATTGGTGCTCAGAACATTACGGGGATTGCTGGGAGGATTGt                                                                                 >  1:764348/1‑301 (MQ=255)`  
                                                                                                                                                                                                                               `catGCCAGGGTATGACCGCCGTGTCAATGGTGGCATCGCCATCATGGGGTCTACTGGTAAAACCACTAAGGAACTTAAGGATGAGATTATGGCTCTAAAAGCGAAAGCTGAGGGAGACGGTAAGAAGACTGGCTAGGTACATGCTTTAATGGATACCGTTAAGGTTCTTACTGTTCGTGCTAGACGCAATCAGGACACTGTGTGGGAAACCTCACTGCGTGCCATCAATGACCTAGGGTTCTTCGCTAAGAACGCCTACATGGGTGCTCAGAACATTACGGAGATTGCCGGGATGATTGTc                                                                                <  1:100199/301‑1 (MQ=255)`  
                                                                                                                                                                                                                                      `gCGTATGACCGCCGTGTCAATGGTGGCATCGCCATCATGGGGTCTACTGGTAAAACCACTAAGGAACTTAAGGATGAGATTTTGGCTCTCAAAGCGAAAGCTGAGGGAGACGGTAAGAAGACTGGCGAGGTACATGCTTTAATGGATACCGTTAAGATTCTTACTGGTCTTGCTAGACGCAATCAGGACACTGTGTGGGAAACCTCACTGCGTGCCATCAATGACCTAGGGTTCTTCGCTAAGAACGCCTACATGGGTGCTCAGAACATTACGGAAATTGCTGGGGTGATTGTCACTGGtt                                                                         >  1:916/1‑300 (MQ=255)`  
                                                                                                                                                                                                                                      `gCGTATGACCGCCGTGTCAATGGTGGCATCGCCATCATGGGGTCTACTGGTAAAACCACTAAGGAACTTAAGGATGAGATTTTGGCTCTCAAAGCGAAAGCTGAGGGAGACGGTAAGAAGACTGGCGAGGTACATGCTTTAATGGATACCGTTAAGATTCTTACTGGTCGTGCTAGACGCAATCAGGACACTGTGTGGGAATCCTCACTGCGTGCCATCAATGACCTAGGGTTCTTCGCTAAGAACGCCTACATGGGTGCTCAGAACATTACGGAGATTGCTGGGATGATTGTCACTGGTa                                                                         >  1:453706/1‑301 (MQ=255)`  
                                                                                                                                                                                                                                      `gCGTATGACCGCCGTGTCAATGGTGACATCGCCATCATGGGGTCTACTGGTAAAACCACTAAGGAACTTAATGATGAGATTTTGGCTCTCAAAGCGAAAGCTGAGGGAGACGGTAAGAAGACTGGCGAGGTACATGCTTTAATGGATACCGTTAAGATTCTTACTGGTCGTGCTAGACGCAATCAGGACACTGTGTGGGAAACCTCACTGCGTGCCATCAATGACCTAGGGTTCTTCGCTAAGAACGCCTACATGGGTTCTCAGAACATTACGGAGATTTCCGGGATGATTGTCAcctgtc                                                                         >  1:576277/1‑296 (MQ=255)`  
                                                                                                                                                                                                                                                `gccgTGTCAATGGTGGCATCGCCATCATGGGGTCTACTGGTAAAACCACTAAGGAACTTAAGGATGAGATTTTGGCTCTCAAAGCGAAAGCTGAGGGAGACGGTAAGAAGACTGGCGAGGTACATGCTTTAATGGATACCGTTAAGATTCTTACTGGTCGTGCTAGACGCAATCAGGACACTGTGTGGGAAACCTCACTGCGTGCCATCAATGACCTAGGGTTCTTCGCTAAGAACGCCTACATGGGTGCTCAGAACATTACGGAGATTGCTGGGGTGATTGTCCCTGGTAACGTTCGttc                                                               >  1:750573/1‑299 (MQ=255)`  
                                                                                                                                                                                                                                                   `gtgtCAATGGTGTCATCGCCATCATGGGGTCTACTGATAAAACCACTAAAGAACTTAAGGATGAGATTTCGCCTCTCAAAGCGAAAGCTGAGGGAGACGGTAAGAAGACTGGCGAGGTACATGATTTAATGGATACCGTTAAGATTCTTACTGGCCGTGCTAGACGCAATCAGTAAACTGTGTGGGAAACCTCACTGCGTGCCATCAATGACCTAGGGTTCTTCGCTAAGAACGCCTACATGGGTGCTCAGAACATTACGGAGATTGCTGGGATGATTGTCACTGGTAACGTTCGTGctct                                                            <  1:459501/301‑1 (MQ=255)`  
                                                                                                                                                                                                                                                    `tgtCAATGGTGGCATCGCCATCATGGTGTCTACTGGTAAAACCACTAAGGAACTTAAGGATGAGATTTTGGCTCTCAAAGCGAAAGCTGAGGGAGACGGTAAGAAGACTGGCGAGGTACATGCTTTAATGGATACCGTTAAGATTCTTACTGGTCGTGCTAGACGCAATAAGGACACTGTGTGGGAAACCTCACTGCGTGCCATCAATGACCTAGGGTTCTTCGCTAAGAACGCCTACATGGGTGCTCAGAACATTACGGAGATTGCTGGGATGATTGTCACTGGTAACGTTCGTGctctt                                                           >  1:77957/1‑300 (MQ=255)`  
                                                                                                                                                                                                                                                         `agacagACATCGCCATCATGGGGTCTACTGGTAAAACCACTAAGGAACTTAAGGATGAGATTTTGGCTCTCCAAGCGAAAGCTGAGGGAGACGGTAAGAAGACTGGCGAGGTACATGCTTTAATGGATACCGTTAAGATTCTTATTGGTCGTGCTAGACGCAATCAGGACACTGTGTGGGAAGCCTCACTGCGTGCCATCAATGACCTAGGGTTCTTCGCTAAGAACGCCTACATGGGTGCTCAGAACATTACGGAGATTGCTGGGATGATTGTCACTGGTAACGTTCGTGCTCTAGGGCa                                                      <  1:568123/296‑1 (MQ=255)`  
                                                                                                                                                                                                                                                          `tGGTGACATCGCCATCATGGGGTCTACTGGTAAAACCACTAAGGAACTTAATGATGAGATTTTGGCTCTCAAAGCGAAAGCTGAGGGAGACGGTAAGAAGACTGGCGAGGTACATGCTTTAATGGATACCGTTAAGATTCTTACTGGTCGTGCTAGACGCAATCAGGACACTGTGTGGGAAACCTCACTGCGTGCCATCAATGACCTAGGGTTCTTCGCTAAGAACGCCTACATGGGTGCTCAGAACATTACGGAGATTGCTGGGATGATTGTCACTGGTAACGTTCGTGCTCTAGGGCAt                                                     >  1:256112/1‑301 (MQ=255)`  
                                                                                                                                                                                                                                                              `ggcATCGCCATCATGGGGTCTACTGGTAAAACCACTAAGGAACTTAAGGATGAGATTTTGGCTCTCAAAGCGAAAGCTGAGGGAGACGGTAAGAAGACTGGCGAGGTACATGCTTTAATGGATACCGTTAAGATTCTTACTGGTCGTGCTAGACGCAATCAGGACACTGTGTGGGAAACCTCACTGCGTGCCATCAATGACCTAGGGTTCTTCGCTAAGAACGCCTACATGGGTGCTCAGAACATTACGGAGATTGCTGGGATGATTGTCACTGATAACGTTCGTGCTCTAGGGCATGGTa                                                 <  1:406487/299‑1 (MQ=255)`  
                                                                                                                                                                                                                                                                       `ataatGTGGTCTACTGGTAAAACCACTAAGGAACTTAAGGATGAGATTTTGGCTCTCAAAGCGAAAGCTGAGGGAGACGGTAAGAAGACTGGCGAGGTACATGCTTTAATGGATACCGTTAAGATTCTTACTGGTAGTGCTAGACGCAATCAGGACACTGTGTGGGAAACCTCACTGCGTGCCATCAATGACCTAGGGTTCTTCGCTAAGAACGCCTACATGGGTGCTCAGAACATTACGGAGATTGCTGGGATGATTGTCACTGGTAACGTTCGTGCTCTAGGGCATGATATCCCAATTc                                        <  1:19933/298‑1 (MQ=255)`  
                                                                                                                                                                                                                                                                        `tcatGGGGTCTACTGGGAAAACCACTAAGGAACTTAAGGATGAGATTTTGGCTCTCAAAGCGAAAGCTGAGGGCAACGGTAAGAAAACAGGCGTGGTACATGCTTTAATGGATACCGTTAAGATTCTTACTGGTCGTGCTAGACGCAATCAGGACACGCTGTGGGAAACATCACTACGTGCCATCAATAACATAGGGTTCTTCACTAAGAACTCCTACATGGGTGCTCAGAACATTACGAAGATTGATGGGATGATTGTCACTGGTAACGTTAGTTCTCTAGGGAATGATATGCCAATTCt                                       >  1:6930/1‑301 (MQ=255)`  
                                                                                                                                                                                                                                                                         `catGGGGTCTACTGGTAAAACCACTAAGGAACTTAAGGATGAGATTTTGGCTATCAAAGCGAAAGCTGAGGGAGACGGTAAGAAGACTGGCGAGGTACATGCTTTAATGGATACCGTTAAGATTCTTACTGGTCGTGCTAGACGCAATCAGGACACTGTGTGGGAAACCTCACTGCGTGCCATCAATGACCTAGGGTTCTTCGCTAAGAACGCCTACATGGGTGCTCAGAACATTACGGAGATTGCTGGGATGATTGTCACTGGTAACGTTCGTGCTCTAGGGCATGGTATCCCAATTCTg                                      >  1:8451/1‑301 (MQ=255)`  
                                                                                                                                                                                                                                                                                             `ccACTAAGGAACTTAATGATGAGATTTTGGCTCTCAAAGCGAAAGCTGAGGGAGACGGTAAGAAGACTGGCGAGGTACATGCTTTAATGGATACCGTTAAGATTCTTACTGGTCGTGCTAGACGCAATCAGGACACTGTGTGGGAAACCTCACTGCGTGCCATCAATGACCTAGGGTTCTTCGCTAAGAACGCCTACATGGGTGCTCAGAACATTACGGAGATTGCCGGGATGATTGTCACTGGTAACGTTCGTGCTCTAGGGCATGGTATCCCAATTCTGCGTGATACACTCTACAAGTc                  >  1:166318/1‑301 (MQ=255)`  
                                                                                                                                                                                                                                                                                                  `aaGGAACTTAAGGATGAGATTTTGGCTCTCCAAGCGAAAGCTGAGGGAGACGGTAAGAAGACTGGCGAGGTACATGCTTTAATGGATACCGTTAAGATTCTTACTGGTCGTGCTAGACGCAATCAGGACACTGTGTGGGAAACCTCACTGCGTGCCATCAATGACCTAGGGTTCTTCGCTAAGAACGCCTACATGGGTGCTCAGAACATTACGGAGATTGCTGGGATGATTGTCACTGGTAACGTTCGTGCTCTAGGGCATGGTATCCCAATTCTGCGTGATACACTCTACAAGTCTAAAc             >  1:224491/1‑301 (MQ=255)`  
                                                                                                                                                                                                                                                                                                   `aGGAACTTAATGATGAGATTTTGGCTCTCAAAGCGAAAGCTGAGGGAGACGGTAAGAAGACTGGCGAGGTACATGCTTTAATGGATACCGTTAAGATTCTTACTGGTCGTGCTAGACGCAATCAGGACACTGTGTGGGAAACCTCACTGCGTGCCATCAATGACCTAGGGTTCTTCGCTAAGAACGCCTACATGGGTGATCAGAACATTACGGAGATTGCCGGGATGATTGTCACTGGTAACGTTCGTGCTCTAGGGCATGGTATCCCAATTCTGCGTGATACACTCTACAAGTCTAAAcc            <  1:398757/301‑1 (MQ=255)`  

                                                                                                                                                                                                                                                                                                             `|`                                                                                                                                                                                                                                                                                                              
`GATGGTAGAGAAGTACGCTATGGATAAGGCTTATGGTATCTCCCACTCAGACCAGTTCACCAACAGTTCCATAATAGAAGAGAACATTGAGGGCTTAGTAGGTATCGAGAATAACTCATTCCTTGAGGCACGTAACTTGTTTGATTCGGACCTATCCATCACTATGCCAGACGGACAGCAATTCTCAGTGAATGACCTAAGGGACTTCGATATGTTCCGCA‑TCATGCCAGCGTATGACCGCCGTGTCAATGGTGACATCGCCATCATGGGGTCTACTGGTAAAACCACTAAGGAACTTAAGGATGAGATTTTGGCTCTCAAAGCGAAAGCTGAGGGAGACGGTAAGAAGACTGGCGAGGTACATGCTTTAATGGATACCGTTAAGATTCTTACTGGTCGTGCTAGACGCAATCAGGACACTGTGTGGGAAACCTCACTGCGTGCCATCAATGACCTAGGGTTCTTCGCTAAGAACGCCTACATGGGTGCTCAGAACATTACGGAGATTGCTGGGATGATTGTCACTGGTAACGTTCGTGCTCTAGGGCATGGTATCCCAATTCTGCGTGATACACTCTACAAGTCTAAACCAGTTTCAGCT  >  NC_001604.1/32769‑33369`

  

Alignment Legend

Aligned base mismatch/match (shaded by quality score): `ATCG/ATCG` `< 3 ≤` `ATCG/ATCG` `< 11 ≤` `ATCG/ATCG` `< 26 ≤` `ATCG/ATCG` `< 37 ≤` `ATCG/ATCG` `< 38 ≤` `ATCG/ATCG`

Unaligned base: `atcg`    Masked matching base: `atcg`    Alignment gap: `‑`    Deleted base: `‑`
